# Editorial: Applications of metabolomics in the formation of food flavor

**DOI:** 10.3389/fnut.2026.1794438

**Published:** 2026-02-18

**Authors:** Wengang Jin, Pengfei Jiang, Xiaohong Sun

**Affiliations:** 1 Qinba State Key Laboratory of Biological Resource and Ecological Environment (Incubation), Shaanxi University of Technology, Hanzhong, China; 2 School of Bioscience and Engineering, Shaanxi University of Technology, Hanzhong, China; 3 State Key Laboratory (SKL) of Marine Food Processing and Safety Control, National Engineering Research Center of Seafood, School of Food Science and Technology, Dalian Polytechnic University, Dalian, China; 4 Department of Plant, Food, and Environmental Sciences, Faculty of Agriculture, Dalhousie University, Truro, NS, Canada

**Keywords:** E-nose, food flavor, GC × GC-TOF MS, GC-IMS, GC-O, metabolomics

Food flavor arises from a complex interplay of metabolites shaped by genetics, processing, and storage (1, 2). Advances in metabolomics now allow detailed mapping of volatile and nonvolatile compounds, revealing how biochemical pathways translate into sensory attributes (3, 4). This Research Topic presents contributions that illustrate how metabolomics provides new insights into food flavor formation across diverse matrices, ranging from meat and poultry to rice, tea, onion, chili powders, plums, and crayfish.

## Insights from animal-based foods

Several articles highlight how metabolomics deepens our understanding of meat quality and flavor. Zhang et al. compared Bian chickens with commercial Cobb broilers and reported that indigenous breeds possess unique protein structures, lipid profiles, and aldehyde-rich volatile compounds linked to richer aromas. Su et al. analyzed Lueyang black-bone chicken meatballs prepared by steaming, boiling, or frying and reported that frying increased key volatiles and sensory appeal. Kang et al. examined crayfish meat and reported that quantitative marination improved umami and flavor nucleotides while lowering biogenic amines, thus enhancing both taste and safety. Together, these studies confirmed the potential value of metabolomics in linking molecular changes to consumer-perceived flavor quality in various foods.

## Plant-based foods and flavor diversity

Metabolomics is equally powerful in plant foods, where secondary metabolites largely define aroma and taste. Cheng et al. profiled pigmented rice and identified seven key metabolites that distinguished varieties with high predictive accuracy through machine learning. For colored onions, the same group detected more than 240 volatiles dominated by sulfur compounds, with random forest models achieving perfect classification accuracy. Hu et al. explored the “Fengtangli” plum, identifying characteristic volatiles such as furan-2-pentyl and (E)-2-octenal that underpin its sweet, citrus-floral notes. These findings demonstrate how metabolomics can support crop breeding, quality evaluation, and product innovation.

## Spices, tea, and aromatic food

Flavor-rich plant products such as spices and tea also benefit from metabolomics-driven analysis. Zhao et al. compared chili powders of different pungency levels and reported distinct volatile profiles and key marker compounds that explain differences in flavor perception. Wang et al. investigated the floral-fruity aroma of Sichuan Congou black tea and identified six aroma-active compounds—including linalool and methyl salicylate—that define its characteristic citrus-like notes. These studies highlight how metabolomics links chemical signatures with consumer-valued sensory traits.

## Cross-cutting advances

Across the contributions, several common themes emerge. The integration of advanced analytical platforms such as GC × GC-TOF MS, HS-GC–IMS, and MDGC-MS/O enables comprehensive metabolite mapping, while coupling these approaches with machine learning supports robust feature selection, classification, and prediction of flavor profiles (5, 6). Importantly, linking sensory evaluation with chemical data strengthens the causal association between metabolite profiles and flavor perception, providing a holistic understanding of flavor development in future (7, 8). In addition, insights into how processing methods—including cooking, marinating, and frying—modulate flavor compounds and influence consumer acceptance highlight the practical applications of metabolomics in food science and industry.

## Future perspectives

This Research Topic demonstrates that various metabolomics have been closely used for flavor research, providing direct tools for sustainable food innovation, biodiversity preservation, and product differentiation. In the future, integration with multi-omics, precision fermentation, and personalized nutrition frameworks will further expand its impact in various food matrix (8–10).

## Conclusion

The collected articles highlight how metabolomics advances our understanding of food flavor across animal- and plant-based products. By linking molecular changes to sensory qualities, metabolomics not only enriches scientific knowledge but also supports innovations in food production, processing, and quality assurance. This Research Topic highlights metabolomics as a cornerstone of modern food science, paving the way for next-generation flavor research. We thank all the contributing authors and reviewers for advancing the field, and we hope that this Research Topic will inspire future explorations at the interface of metabolomics, food science, and consumer preferences.
